# Concurrent Activation of Acetylation and Tri-Methylation of H3K27 in a Subset of Hepatocellular Carcinoma with Aggressive Behavior

**DOI:** 10.1371/journal.pone.0091330

**Published:** 2014-03-10

**Authors:** Akimasa Hayashi, Naoko Yamauchi, Junji Shibahara, Hiroshi Kimura, Teppei Morikawa, Shumpei Ishikawa, Genta Nagae, Akihiro Nishi, Yoshihiro Sakamoto, Norihiro Kokudo, Hiroyuki Aburatani, Masashi Fukayama

**Affiliations:** 1 Department of Pathology, Graduate School of Medicine, The University of Tokyo, Tokyo, Japan; 2 Genome Science Division, Research Center for Advanced Science and Technology (RCAST), The University of Tokyo, Tokyo, Japan; 3 Biomolecular Networks Laboratories Group, Graduate School of Frontier Biosciences, Osaka University, Suita, Japan; 4 Department of Genomic Pathology, Medical Research Institute, Tokyo Medical and Dental University, Tokyo, Japan; 5 Hepato-Biliary-Pancreatic Surgery Division, Department of Surgery, Graduate School of Medicine, The University of Tokyo, Tokyo, Japan; 6 Yale Institute for Network Science, New Haven, Connecticut, United States of America; Kanazawa University, Japan

## Abstract

Analysis of acetylation and tri-methylation of the same residue of histone molecules might identify a subset of hepatocellular carcinoma (HCC) with aggressive behavior. In the present study, we examined acetylation and tri-methylation of lysine 27 on histone H3 (H3K27ac and H3K27me3, respectively) because these two modifications are known to exhibit opposite effects (enhancing and silencing) on gene expression. Neoplastic and non-neoplastic tissues from 198 HCC cases were immunostained with specific monoclonal antibodies against H3K27ac and H3K27me3. The stained tissues were evaluated by an image analyzing program to generate histological scores (H-scores, range 0–300), which were determined by multiplying the percentage of positive-stained cells with the classified immunohistochemical marker intensity (0–3). HCC tissues showed significantly higher H3K27ac (156.7±86.8) and H3K27me3 H-scores (151.8±78.1) compared with the background liver (40.3±33.0 and 64.7±45.6, respectively) (both *P*<0.001). The cases with H-scores of high-H3K27ac/high-H3K27me3 (n = 54) showed significant correlation with poor differentiation of morphology (*P*<0.01) and p53-positive staining (*P*<0.05), and poor prognosis (*P*<0.01). Confocal microscopy revealed segregated intranuclear localization of both modifications in the individual cancer cells: H3K27ac localization in central euchromatin regions and H3K27me3 in peripheral heterochromatin regions. Concurrent acetylation and methylation at H3K27 occurs in HCC cells in association with p53 abnormalities. These findings demonstrate that image analyzer-assisted H-scores of H3K27ac and H3K27me3 identified an aggressive subgroup of HCC, and could serve as a prognostic marker for HCC.

## Introduction

Hepatocellular carcinoma (HCC) is one of the major cancers worldwide [1]. HCC is especially common in Asia-Pacific countries and is ranked the fourth highest cause of death among cancers in Japan [2]. Despite recent advances in resection and ablation techniques, the recurrence rate after initial treatment is high and prognosis is poorer than other carcinomas [2], [3]. Improved risk stratification and accurate individualized prediction of postoperative recurrence and survival can help guide patient counseling, follow-up scheduling, administration of adjuvant therapies, and design of clinical trials [4].

Accumulating evidence has shown that not only genetic but also epigenetic changes play crucial roles in the genesis and prognosis of cancer [5], [6]. Global levels of several histone modifications, as well as histone modification enzymes, have clinical significance in several cancers [7]–[9]. A recent review on histone modifications and cancer also referred its potential that serves as a biomarker [10]. Previous studies in HCC demonstrated the clinical significance of individual histone methylation levels. High levels of tri-methylation of lysine 4 on histone H3 (H3K4me3) and tri-methylation of lysine 27 on histone H3 (H3K27me3) correlated with aggressive features and poor prognosis [11], [12]. However, little is known about global histone acetylation levels in HCC. One immunohistochemical study revealed that the levels of acetylation of lysine 9 on histone 3 (H3K9ac) and acetylation of lysine 8 on histone 4 (H4K8ac) were higher in HCC than in non-cancerous liver, but the clinical significance remains unknown [13].

In this study, we focused on acetylation of lysine 27 on histone H3 (H3K27ac) and its relation with H3K27me3. H3K27ac is an active enhancer marker and reflects global cell-type-specific gene expression in various cancer cell lines [14]. H3K27me3 is another histone modification of the same site, and acts instead as a silencer [15]. We evaluated both H3K27ac and H3K27me3 levels in HCC using specific monoclonal antibodies, and used digital slide scanner and image analyzing software to quantify the results as objectively as possible [16]. In addition, we examined nuclear localization of H3K27ac and H3K27me3 by double immunofluorescence in frozen sections. In evaluating the clinicopathological significance, we also assessed the status of p53 and β-catenin, two major HCC driver genes [17], because interplay between the cancer genome and epigenome is important, especially in HCC [6].

## Materials and Methods

### Ethics Statement

This study was approved by the University of Tokyo Institutional Ethical Committee. Clinical samples with written informed consent were collected under the University of Tokyo Institutional guidelines for the study of human tissues.

### Tissue microarrays

A total of 198 primary HCC cases were retrieved from the archives of the Department of Pathology of The University of Tokyo Hospital to generate tissue microarrays (TMAs). Surgical resections were performed between 1995 and 2006 based on Makuuchi criterion for resection of HCC [18]. Resection samples were fixed with formalin and embedded in paraffin. Hematoxylin and eosin (H&E)-stained slides of all the cases were reviewed. Histological diagnosis was based on the most recent criteria proposed by the Liver Cancer Study Group of Japan [19]. TMAs were generated according to well-established procedures [20]. In brief, two tissue cores (2 mm diameter each) were punched out of each donor paraffin block and transferred to each of the recipient TMA blocks. TMAs for the paired background livers were also produced.

### Frozen samples

Frozen samples were used for western blotting and double immunofluorescent immunohistochemistry. Fresh resection specimens were embedded in Tissue-Tek OCT compound (Sakura Finetek, Torrance, CA, USA) and frozen in a dry-ice acetone bath. Samples were kept at −80°C until analysis.

### Immunohistochemistry

Whole tissue and TMA blocks were sectioned in 4 µm thickness and stained with a mouse monoclonal antibody against H3K27ac generated by one of the authors (CMA309; Hiroshi Kimura Laboratory; 1 µg/mL) and rabbit monoclonal antibody against H3K27me3 (#9733; Cell Signaling Technology, Beverly, MA, USA; 1∶200 dilution). The reliability and specificity of the anti-H3K27ac antibody were examined in a previous study [21]. Immunohistochemical staining was performed according to standard techniques on a Ventana Benchmark XT autostainer (Ventana Medical Systems Inc., Tucson, AZ, USA). The appropriate positive and negative controls were included.

Whole section slides of all cases were also immunostained with anti-p53 antibody (NCL-p53-505; Novocastra Laboratories, Newcastle, UK; 1∶50 dilution) and anti-β-catenin antibody (610154; BD Transduction Laboratories, Lexington, KY, USA; 1∶300 dilution) using the same protocols.

### Western blotting

Frozen samples were sliced and lysed in lysis buffer (10 mmol/L Tris-HCl, pH 7.4, 150 mmol/L NaCl, 5 mmol/L EDTA, 1.0% Triton X-100, 1.0% sodium deoxycholate, 0.1% SDS, and 1 mmol/L of phenylmethylsulfonyl fluoride with protease inhibitor cocktail). Protein samples were loaded (10 µg of protein per lane) and fractionated on a 12% SDS polyacrylamide gel. After transfer onto a polyvinylidene difluoride membrane, membranes were probed with anti-H3K27ac (0.4 µg/mL) and anti-H3K27me3 (1∶1000 dilution) antibodies. The membranes were visualized using the ECL Plus Western Blotting Detection System (GE Healthcare UK, Little Chalfont, UK). Hybridization with anti actin-β antibody (A5441; Sigma, Munich, Germany; 1∶20000) was used as a loading control.

### Evaluation of immunohistochemistry

**Figure 1:**
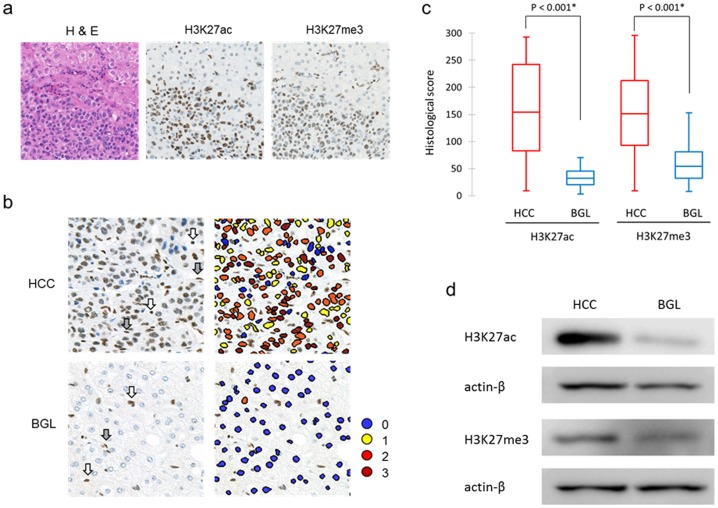
H3K27ac and H3K27me3 in HCC and background liver (BGL). (a) Images of hematoxylin and eosin (H&E), H3K27ac and H3K27me3 staining around the border between HCC and BGL. (b) Images of HCC and BGL samples by objective-quantitative analysis with Tissue Studio (Definiens AG, Munich, Germany). Each nucleus was assigned scores of 0 (negative), 1 (weakly positive), 2 (moderately positive) or 3 (strongly positive). Inflammatory cells (white arrows) and endothelial cells (gray arrows) were excluded in this analysis. (c) Distributions of histological scores (H-scores) for H3K27ac and H3K27me3 in HCCs and BGLs using box-plots. Statistical analyses were performed using the Mann-Whitney test. (d) Western blot showing increased levels of H3K27ac and H3K27me3 in HCCs compared with the matched non-cancerous liver. Actin-β was used as a loading control.

After all slides were digitalized with a digital slide scanner (Nano Zoomer, Hamamatsu Photonics, Shizuoka, Japan), immunohistochemical results were scored with an image analyzing program (Tissue Studio, Definiens AG, Munich, Germany) [16]. Lymphocytes were used as internal positive controls in each case. Histological scores (H-scores) were determined as discussed in previous studies [22]–[24]. In brief, the H-score was calculated by multiplying the percentage of positive cells (0 to 100%) by the classified immunohistochemical marker intensity (0 to 3; as shown in **Figure 1a**). The H-scores ranged from 0 to 300. Most of the inflammatory cells and endothelial cells were excluded in this analysis by size and geometry selection of the analyzing program.

Intranuclear p53 or β-catenin positive staining was judged as positive/negative, according to standards described in the previous report [25].

### Double immunofluorescent immunohistochemistry

To examine the localization of H3K27ac and H3K27me3 in nuclei, we performed double immunofluorescent immunohistochemistry in frozen samples. Whole tissue sections were incubated for 2 hours at room temperature with rabbit monoclonal anti-H3K27me3 antibody (1∶200 dilution) along with mouse monoclonal anti-H3K27ac antibody (2 µg/mL). After washes, sections were incubated with Alexa Fluor 488 goat anti-mouse IgG (Invitrogen, Molecular Probes, Carlsbad, CA, USA; 1∶1000) and Alexa Fluor 594 goat anti-rabbit IgG (Invitrogen; 1∶1000) at room temperature for 1 hour. The immunolabeled frozen sections were observed with a Leica TCS SP5 spectral scanning confocal microscope (Leica Microsystems, Mannheim, Germany). Captured images of immunolabeled frozen sections were analyzed by Leica Application Suite (LAS) (Leica Microsystems, Mannheim, Germany), as previously described [26].

### Statistical analysis

All statistical analyses except the time-dependent receiver operating characteristic (ROC) analysis were performed using JMP Pro 10 (SAS Institute Inc., Cary, NC, USA). Differences were considered significant at *P*<0.05. Categorical data were analyzed using two-sided Fisher's exact test. The Kaplan-Meier method and log-rank test were used to analyze survival. For multi-group comparison in the Kaplan-Meier method, the Bonferroni adjustment was used [27]. Multivariate Cox proportional hazards regression models were used to control for confounding variables. The multivariate regression analysis was performed using the significant clinicopathological variables identified in the univariate analysis. The variable “pathological stage” was excluded in multivariate analysis because pathological stage was determined by other pathological variables, such tumor size and number and vascular invasion [19]. The time-dependent ROC analysis was performed to evaluate the discriminatory ability for the prediction of survival using R software (version 3.0.2, http://www.r-project.org) and its specific package as described previously [28], [29]. The 5-year overall and recurrence-free models were used to perform this analysis and the area under the curve (AUC) was calculated for measuring the prediction accuracy [30].

## Results

### Immunohistochemical analysis of H3K27ac and H3K27me3 in HCC

Immunohistochemical analyses of H3K27ac and H3K27me3 revealed positive staining of both in nuclei of HCC (**Figure 1a and 1b**). Approximately 40% of cases showed over 80% of cells containing vivid positive staining (IHC marker intensity 2–3, H-score >200), and 40% of cases showed various nuclear staining levels (IHC marker intensity 1–3, H-score 100–200). In the remaining 20% of cases, less than 50% cells exhibited weak or moderate positive staining (IHC marker intensity 1–2, H-score<100). As for the background liver, in over 80% of cases, few cells exhibited weak or moderate positive staining (IHC marker intensity 1–2, H-score<50). In total, the H-score of H3K27ac was 156.7±86.8 (mean±standard deviation [SD], range: 9–293) in HCC and 40.3±33.0 (range: 3–159) in background liver. The H-score of H3K27me3 was 151.8±78.1 in HCC (range: 9–296) and 64.7±45.6 (range: 8–211) in background liver. Mann–Whitney test showed that H-scores of H3K27ac and H3K27me3 were higher in HCC than those in background livers (*P*<0.001) (**Figure 1c**). Western blotting analysis of frozen samples confirmed that H3K27ac and H3K27me3 levels were elevated in HCC (**Figure 1d**).

### Distributions of H3K27ac and H3K27me3 in HCC

**Figure 2:**
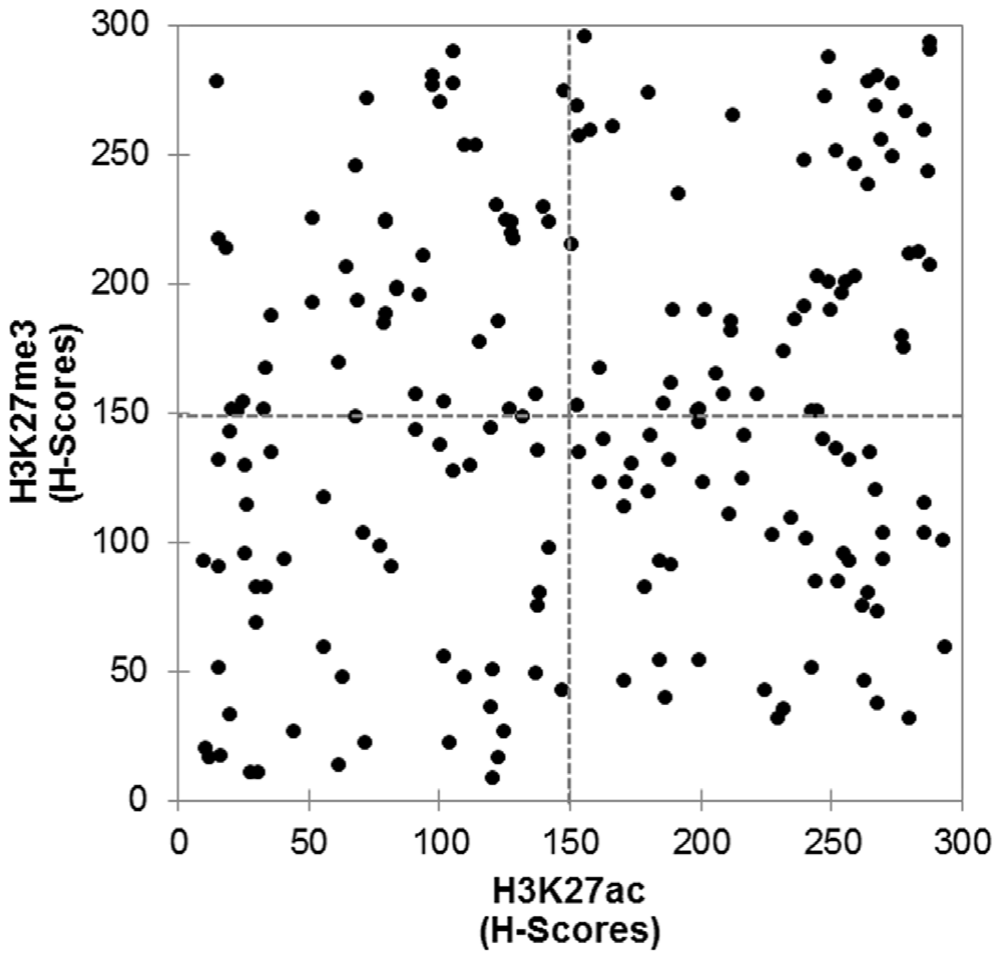
Distributions of histological scores (H-scores) of H3K27ac and H3K27me3 in HCC. H-score of 150 is shown in dashed lines.

Distributions of H3K27ac and H3K27me3 H-scores are shown in **Figure 2**. There was no significant correlation between these two modifications (r = 0.165). Cases were divided into two groups based on the H-scores, as in the previous study on H3K27 modification [24]: the low H-score group (H-score ≤150) and the high H-score group (H-score >150).

### H3K27 modification and correlation with clinicopathological characteristics

**Figure 3:**
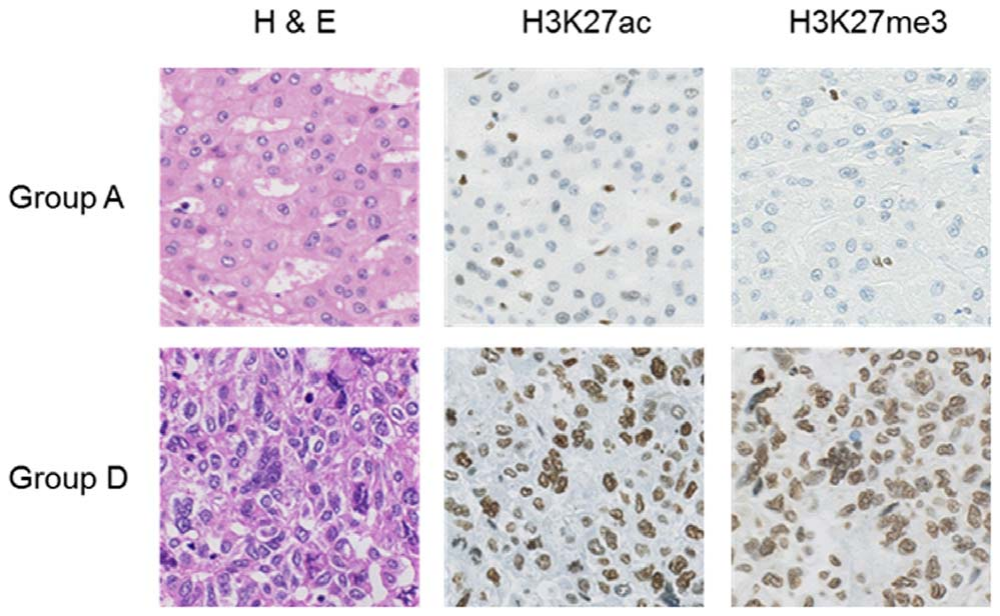
Images of H&E staining and H3K27ac and H3K27me3 immunohistochemistry of Group A (low-H3K27ac/low-H3K27me3) and Group D cases (high-H3K27ac/high-H3K27me3).

Based on the two-category classification (low/high H-score groups), the high H3K27me3 group correlated with poor differentiation, vascular invasion and serum alpha-fetoprotein (AFP) level, and the high H3K27ac group also had association with poor tumor differentiation (**Table S1**). To investigate the significance of concurrent activation of both histone modifications at the same residue of H3 in more detail, the cases were re-classified into four groups (Group A–D). Group A included cases with low H-scores for both H3K27ac and H3K27me3 (low-H3K27ac/low-H3K27me3 group), Group B included high-H3K27ac/low-H3K27me3 cases, Group C included low-H3K27ac/high-H3K27me3 cases, and Group D included high-H3K27ac/high-H3K27me3 cases. Representative photomicrographs of H3K27ac and H3K27me3 immunohistochemistry of Group A and Group D cases are shown in **Figure 3**.

The correlation of clinicopathological variables with the four groups (Group A–D) revealed that Group D (high-H3K27ac/high-H3K27me3 group) had a strong association with poor differentiation (**Table 1**). Group D also showed significant correlation with p53-positive staining (**Table 2** **, Figure S1**). The levels of β-catenin staining were not different among the four groups.

**Table 1:** Correlation between H3K27 modification and clinicopathological features in patients with HCC who underwent hepatectomy.

Clinical or pathological feature	Total N	H3K27 modification group				*P* value
		A	B	C	D	
All cases	198	49	49	46	54	
Age (years)						0.770
≤60	64	16	17	12	19	
>60	134	33	32	34	35	
Sex						0.831
Male	152	40	37	34	41	
Female	46	9	12	12	13	
HBs Ag						0.913
Positive	45	11	11	9	14	
Negative	153	38	38	37	40	
HCV Ab						0.435
Positive	132	31	37	31	33	
Negative	66	18	12	15	21	
Cirrhosis						0.441
Yes	113	28	28	22	35	
No	85	21	21	24	19	
Pathological stage						0.185
Stage 1	18	5	6	5	2	
Stage 2	86	24	25	16	21	
Stage 3	74	14	14	18	28	
Stage 4a	20	6	4	7	3	
Maximum tumor size (mm)						0.356
≤50	144	40	33	34	37	
>50	54	9	16	12	17	
Histological grade						0.003*
well	52	19	14	13	6	
mod	115	27	30	27	31	
por	31	3	5	6	17	
No. of lesions						0.603
1	130	30	35	32	33	
≥2	68	19	14	14	21	
Vascular invasion						0.051
Yes	64	12	11	21	20	
No	134	37	38	25	34	
Serum AFP level (ng/mL)						0.104
≤200	136	39	36	29	32	
>200	62	10	13	17	22	
Serum DCP level (mAU/mL)						0.082
≤100	111	35	25	22	29	
>100	87	14	24	24	25	

AFP: alpha-fetoprotein DCP: des-gamma-carboxy prothrombin.

**Table 2:** Correlation between H3K27 modification and nuclear accumulation of p53 and β-catenin in patients with HCC who underwent hepatectomy.

Molecular feature	Total N	H3K27 modification group				*P* value
		A	B	C	D	
All cases	198	49	49	46	54	
p53						0.032*
Positive	50	9	10	9	22	
Negative	148	40	39	37	32	
β-catenin						0.312
Positive	43	11	7	9	16	
Negative	155	38	42	37	38	

**Figure 4:**
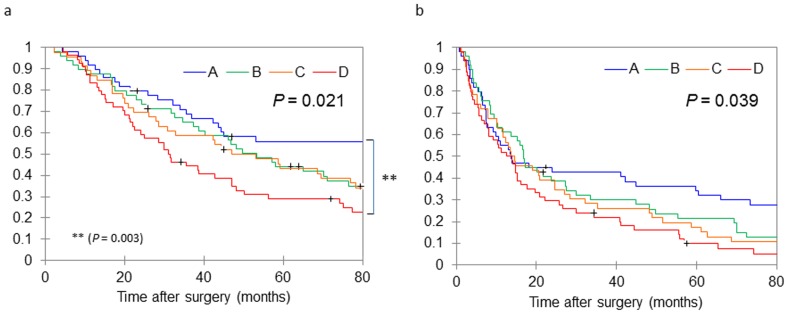
Kaplan–Meier analysis of (a) overall survival and (b) recurrence-free survival after resection according to histone H3K27 modification in HCC. Log-rank analysis showed significant differences in overall survival among the four groups (A–D) in both overall (*P* = 0.021) and recurrence free (*P* = 0.039) survival. Censored data were plotted as “+” in the Kaplan-Meier curve.

The median follow-up period was 45.0 months (range 2.3–196.2 months). The overall cumulative survival rate at five years was 40.4% and the recurrence free cumulative survival rate at five years was 19.2%. Kaplan–Meier analysis revealed significant difference of overall and recurrence-free survival in the four H3K27 modification groups in HCC (log-rank *P* = 0.021 and *P* = 0.039, respectively) (**Figure 4**). Group D showed significantly poorer prognosis in overall survival than Group A (*P* = 0.003) even after the Bonferroni correction for multi-group comparison (**Table S2**). The univariate Cox regression analysis also showed that Group D had significantly poorer prognosis than Group A both in overall survival and in recurrence free survival (**Table 3**). In addition, the multivariate Cox regression analysis revealed that Group D had significant poorer prognosis than Group A in overall survival (**Table 4**).

**Table 3:** H3K27 modification in HCC and patient outcome (univariate analysis).

	Overall survival					Recurrence free survival				
	HR	95% CI			*P*-value	HR	95% CI			*P*-value
Age >60 years (vs ≤60)	1.378	0.955	–	1.988	0.087	1.247	0.902	–	1.724	0.182
Sex female (vs male)	1.255	0.863	–	1.827	0.235	1.034	0.729	–	1.468	0.851
HBs Ag positive (vs negative)	0.951	0.632	–	1.432	0.811	1.065	0.741	–	1.533	0.733
HCV Ab positive (vs negative)	1.204	0.840	–	1.727	0.312	1.233	0.893	–	1.704	0.204
Cirrhosis positive (vs negative)	1.804	1.275	–	2.554	<0.001*	1.529	1.127	–	2.075	0.006*
Pathological stage					<0.001*					<0.001*
Stage 2 (vs Stage 1)	1.948	0.885	–	4.289	0.098	1.334	0.750	–	2.374	0.327
Stage 3 (vs Stage 1)	4.683	2.131	–	10.29	<0.001*	3.281	1.838	–	5.857	<0.001*
Stage 4 (vs Stage 1)	6.286	2.644	–	14.94	<0.001*	3.543	1.778	–	7.061	<0.001*
Maximum tumor size >50 mm (vs ≤50)	1.517	1.060	–	2.173	0.023*	1.691	1.217	–	2.349	0.002*
Histological grade					0.011*					0.011*
mod (vs well)	1.149	0.769	–	1.716	0.497	1.146	0.803	–	1.635	0.453
por (vs well)	2.186	1.309	–	3.649	0.004*	2.009	1.261	–	3.200	0.003*
No. of Tumor ≥2 (vs 1)	2.219	1.580	–	3.114	<0.001*	1.985	1.451	–	2.717	<0.001*
Vascular invasion positive (vs negative)	2.592	1.843	–	3.644	<0.001*	2.587	1.887	–	3.548	<0.001*
Serum AFP level >200 ng/mL (vs ≤200)	1.697	1.198	–	2.380	0.003*	1.845	1.301	–	2.591	<0.001*
Serum DCP level >100 mAU/mL (vs ≤100)	1.140	0.817	–	1.586	0.438	1.206	0.863	–	1.678	0.270
p53 positive (vs negative)	1.516	1.053	–	2.183	0.025*	1.502	1.072	–	2.104	0.018*
β-catenin positive (vs negative)	1.343	0.909	–	1.986	0.139	1.345	0.942	–	1.920	0.103
H3K27 modification					0.023*					0.040*
Group B (vs Group A)	1.347	0.820	–	2.213	0.239	1.275	0.822	–	1.980	0.278
Group C (vs Group A)	1.519	0.924	–	2.499	0.099	1.537	0.986	–	2.395	0.058
Group D (vs Group A)	2.054	1.285	–	3.284	0.003*	1.823	1.186	–	2.802	0.006*

CI, confidence interval; HR, hazard ratio.

**Table 4:** H3K27 modification in HCC and patient outcome (multivariate analysis).

	Overall survival					Recurrence free survival				
	HR	95% CI			*P*-value	HR	95% CI			*P*-value
Cirrhosis positive (vs negative)	1.903	1.322	–	2.775	<0.001*	1.828	1.316	–	2.561	<0.001*
Maximum tumor size >50 mm (vs 50≥)	1.239	0.799	–	1.895	0.334	1.488	0.998	–	2.189	0.051
Histological grade					0.095					0.111
mod (vs well)	0.769	0.491	–	1.218	0.259	0.808	0.545	–	1.211	0.299
por (vs well)	1.261	0.674	–	2.337	0.466	1.279	0.735	–	2.204	0.381
No. of Tumor ≥2 (vs 1)	2.095	1.440	–	3.040	<0.001*	1.763	1.250	–	2.474	0.001*
Vascular invasion positive (vs negative)	2.130	1.416	–	3.186	< 0.001*	2.212	1.502	–	3.235	<0.001*
Serum AFP level >200 ng/mL (vs ≤200)	1.184	0.776	–	1.788	0.429	1.038	0.697		1.527	0.851
p53 positive (vs negative)	1.072	0.703	–	1.606	0.743	1.206	0.822	–	1.742	0.333
H3K27 modification					0.171					0.686
Group B (vs Group A)	1.492	0.898	–	2.494	0.122	1.270	0.809	–	2.000	0.299
Group C (vs Group A)	1.546	0.907	–	2.652	0.101	1.253	0.783	–	2.010	0.347
Group D (vs Group A)	1.730	1.037	–	2.925	0.036*	1.275	0.790	–	2.073	0.321

CI, confidence interval; HR, hazard ratio.

The time-dependent ROC analysis showed that the H3K27 modification can predict 5-year recurrence-free survival (AUC = 0.662) especially in early-stage HCC (stage 1, 2) (AUC = 0.712) (**Figure S2**). The AUC of H3K27 modification is higher than other prognostic indicators or markers previously reported [31], such as AFP (AUC = 0.615 and AUC = 0.583, in all stage and in early-stage HCC, respectively). In the 5-year overall survival model, AUCs of H3K27 modification were nearly the same as those of other predictors.

### Immunofluorescent analysis of H3K27ac and H3K27me3 in HCC

**Figure 5:**
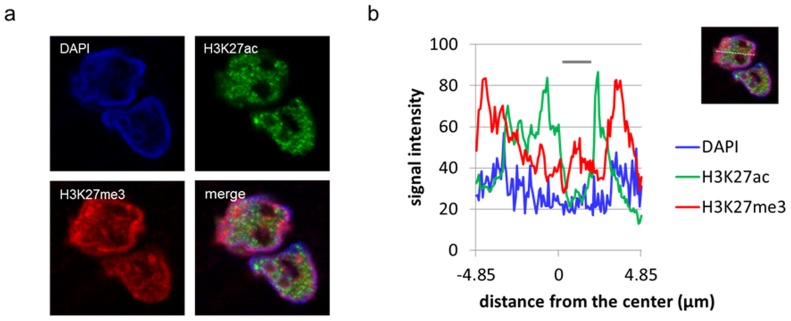
Double immunofluorescent staining of H3K27ac and H3K27me3 in HCC. (a) Images of double immunofluorescent staining of H3K27ac and H3K27me3 in frozen samples. (b) Distribution of immunofluorescent signal intensity in nucleus. Image analysis was performed of the dashed line shown in the merged image using LAS (Leica Microsystems, Mannheim, Germany). Nucleolus regions are shown by the gray bar.

The images of double immunofluorescence staining of H3K27ac and H3K27me3 in frozen sections are shown in **Figure 5a**. These two modifications were detected as mutually exclusive dot-like regions in the nucleus. H3K27ac tended to localize in the central euchromatin region of nuclei, whereas H3K27me3 was observed in the peripheral heterochromatin zone. Different immunofluorescent intensity peaks between H3K27ac and H3K27me3 were confirmed by quantitative intensity analysis using image analyzing software (**Figure 5b**).

## Discussion

This is the first study that evaluated the clinicopathological significance of concurrent increase of H3K27ac and H3K27me3 in HCC. Although both modifications appeared to occur independently, concurrent activation of acetylation and methylation at the same residue, H3K27, was observed in a subset of HCC with poorly-differentiated morphology and p53 abnormality, and the patients with this subset of HCC showed poor prognosis compared with others. These results could not be obtained without the precise quantification of immunohistochemical results in the present study. We confirmed that the system is feasible and reproducible [16]. The obtained data represented very well subtle differences among the cases, as illustrated in the scatter diagram (**Figure 2**).

Additionally, both modifications showed different nuclear localization patterns in individual cancer cells; H3K27ac was localized in the central euchromatin regions, while H3K27me3 was observed in the peripheral heterochromatin regions [32]. H3K27ac acts as an active enhancer, while H3K27me3 acts as a silencer [15]. Thus, the segregated localization might reflect compartmentalization of oncogenes related to proliferation or invasion and tumor suppressor genes related to apoptosis or differentiation within the nucleus [33], [34].

H3K27ac modification is induced by p300/CBP and removed by HDACs, while H3K27me3 is induced by EZH2 and removed by JMJD3 [6]. Previous studies revealed the clinicopathological significance of expression of these modification enzymes. High expression of p300 in HCC correlated with poor differentiation and poor prognosis and high expression of EZH2 in HCC correlated with poor differentiation [35]–[38]. As for H3K27ac, high expressions of some HDACs in HCC also correlated with de-differentiation and worse survival [39], [40]. These data are consistent with our results, but further studies are necessary to evaluate which targets are better for the patient stratification, either the modifying enzymes or the histone modifications.

The HCC subgroup with concurrently high H-scores of H3K27ac and H3K27me3 showed significant correlation with positive staining of p53. Positive p53 staining has been noted to correlate with p53 mutation in HCC [41]. The correlation of p53 abnormality with p300 and EZH2 has been recognized in several cancers. Nuclear accumulation of p53 was associated with p300 expression in breast cancer [42] and with EZH2 expression in squamous cell carcinoma of esophagus [43]. In an *in vitro* study, overexpression of mutant p53 upregulated EZH2 expression [44]. Di Agostino et al. demonstrated that mutant p53 is able to recruit p300 and cause histone acetylation in some promoter regions [45]. Therefore, some of the p53 abnormalities might cause concurrent increase of H3K27ac and H3K27me3 levels through mechanisms that have not been investigated yet.

In summary, concurrent activation of acetylation and methylation on H3K27 occurs in the subgroup of HCC, showing p53-positive poorly differentiated type. Further studies are necessary to evaluate the possible association between p53 and the concurrent increase of histone modification. Our findings demonstrate that image analyzer-assisted H-scores of H3K27ac and H3K27me3 identified an aggressive subgroup of HCC, and could serve as a prognostic marker for HCC. Additional ROC analysis supports the possibility that the H3K27 modification might predict more accurately in HCC than other prognostic indicators or markers.

## Supporting Information

Figure S1 **Representative photomicrographs of p53 and β-catenin immunohistochemistry.**
(TIF)

Figure S2 **Time-dependent ROC analysis using (a) 5-year overall survival and (b) 5-year recurrence-free survival models in HCC.** Prognostic clinical and pathological valuables, tumor markers and H3K27 modification were entered; cirrhosis (positive or negative), tumor size (mm), tumor number (number), histological grade (well, moderate or poor), vascular invasion (positive or negative), AFP (ng/mL), DCP (mAU/mL), p53 (positive or negative), β-catenin (positive or negative) and H3K27 (group A, B, C or D). 95% confidence intervals were shown after AUC values.
(TIF)

Table S1 **Correlation between H3K27ac and H3K27me3 with clinicopathological features in patients with HCC who underwent hepatectomy.**
(DOC)

Table S2 **Multiple comparison in H3K27 modification groups using the log-rank test.**
(DOC)
