# Supplementary figures and images for: Concurrent Activation of Acetylation and Tri-Methylation of H3K27 in a Subset of Hepatocellular Carcinoma with Aggressive Behavior
Source: PLoS One. 2014 Mar 10;9(3):e91330. doi: 10.1371/journal.pone.0091330 (PMC3948868; doi:10.1371/journal.pone.0091330)

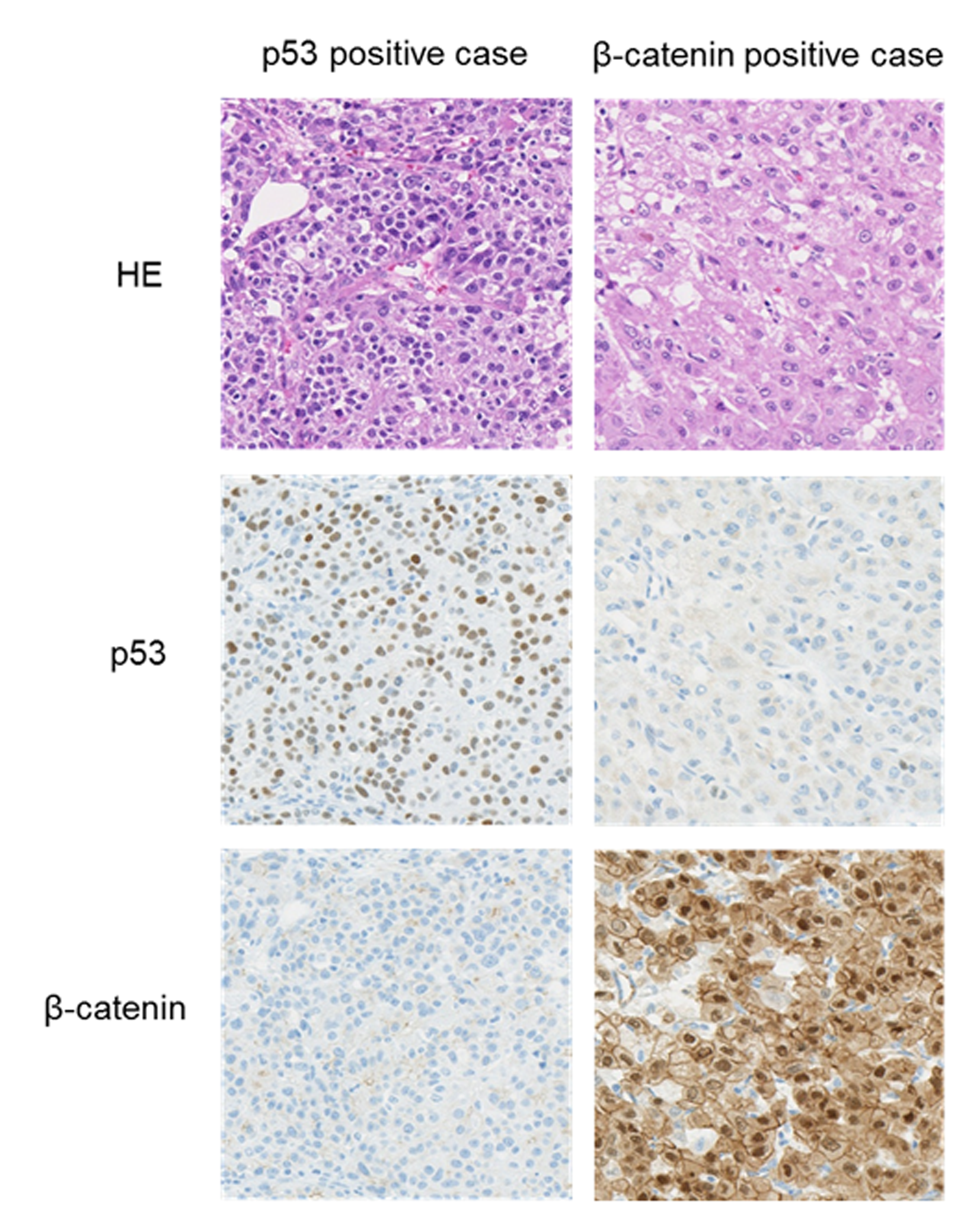

Supplement: Figure S1 — Representative photomicrographs of p53 and β-catenin immunohistochemistry. (TIF) [file pone.0091330.s001.tif]

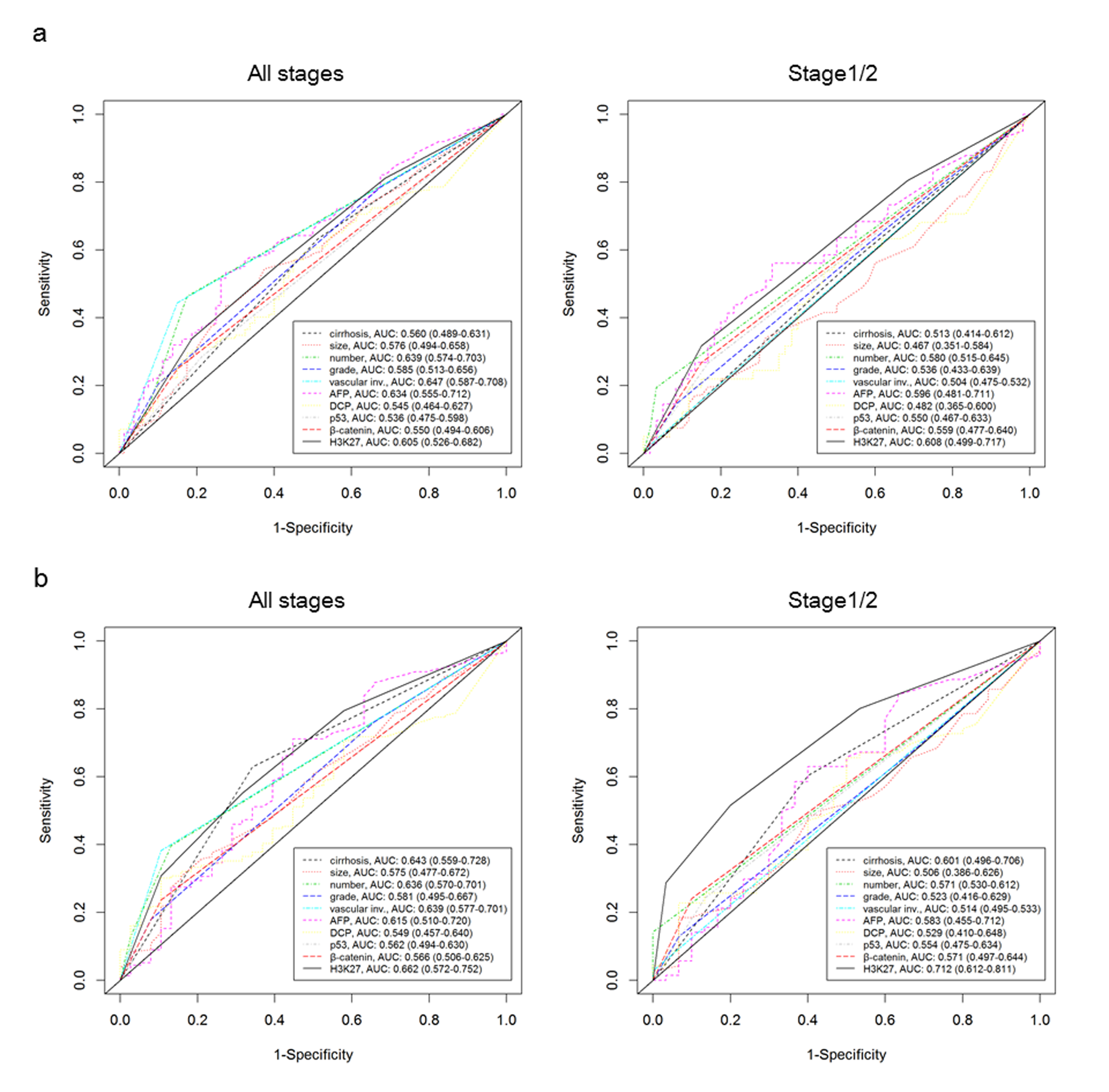

Supplement: Figure S2 — Time-dependent ROC analysis using (a) 5-year overall survival and (b) 5-year recurrence-free survival models in HCC. Prognostic clinical and pathological valuables, tumor markers and H3K27 modification were entered; cirrhosis (positive or negative), tumor size (mm), tumor number (number), histological grade (well, moderate or poor), vascular invasion (positive or negative), AFP (ng/mL), DCP (mAU/mL), p53 (positive or negative), β-catenin (positive or negative) and H3K27 (group A, B, C or D). 95% confidence intervals were shown after AUC values. (TIF) [file pone.0091330.s002.tif]
