# Supplementary material for: Concurrent Activation of Acetylation and Tri-Methylation of H3K27 in a Subset of Hepatocellular Carcinoma with Aggressive Behavior
Source: PLoS One. 2014 Mar 10;9(3):e91330. doi: 10.1371/journal.pone.0091330 (PMC3948868; doi:10.1371/journal.pone.0091330)
Supplement: Table S1 — Correlation between H3K27ac and H3K27me3 with clinicopathological features in patients with HCC who underwent hepatectomy. (DOC) [file pone.0091330.s003.doc]

**Table S1.** Correlation between H3K27ac and H3K27me3 with clinicopathological features in patients with HCC who underwent hepatectomy.

| Clinical, pathological or molecular feature | Total N | H3K27ac | | *P* value |  | H3K27me3 | | *P* value |
| --- | --- | --- | --- | --- | --- | --- | --- | --- |
|  |  | staining | |  |  | staining | |  |
|  |  | low | high |  |  | low | high |  |
| All cases | 198 | 95 | 103 |  |  | 98 | 100 |  |
|  |  |  |  |  |  |  |  |  |
| Age |  |  |  | 0.449 |  |  |  | 0.762 |
| ≤ 60 | 64 | 28 | 36 |  |  | 33 | 31 |  |
| > 60 | 134 | 67 | 67 |  |  | 65 | 69 |  |
| Sex |  |  |  | 0.739 |  |  |  | 0.615 |
| Male | 152 | 74 | 78 |  |  | 77 | 75 |  |
| Female | 46 | 21 | 25 |  |  | 21 | 25 |  |
| HBs Ag |  |  |  | 0.615 |  |  |  | 1.000 |
| Positive | 45 | 20 | 25 |  |  | 22 | 23 |  |
| Negative | 153 | 75 | 78 |  |  | 76 | 77 |  |
| HCV Ab |  |  |  | 0.763 |  |  |  | 0.453 |
| Positive | 132 | 62 | 70 |  |  | 68 | 64 |  |
| Negative | 66 | 33 | 33 |  |  | 30 | 36 |  |
| Cirrhosis |  |  |  | 0.252 |  |  |  | 1.000 |
| Yes | 113 | 50 | 63 |  |  | 56 | 57 |  |
| No | 85 | 45 | 40 |  |  | 42 | 43 |  |
| Pathological stage |  |  |  | 0.331 |  |  |  | 0.071 |
| Stage 1 | 18 | 10 | 8 |  |  | 11 | 7 |  |
| Stage 2 | 86 | 40 | 46 |  |  | 49 | 37 |  |
| Stage 3 | 74 | 32 | 42 |  |  | 28 | 46 |  |
| Stage 4a | 20 | 13 | 7 |  |  | 10 | 10 |  |
| Maximum tumor size (mm) |  |  |  | 0.150 |  |  |  | 0.634 |
| ≤ 50 | 144 | 74 | 70 |  |  | 73 | 71 |  |
| > 50 | 54 | 21 | 33 |  |  | 25 | 29 |  |
| Histological grade |  |  |  | 0.016* |  |  |  | 0.004* |
| well | 52 | 32 | 20 |  |  | 33 | 19 |  |
| mod | 115 | 54 | 61 |  |  | 57 | 58 |  |
| por | 31 | 9 | 22 |  |  | 8 | 23 |  |
| No. of lesions |  |  |  | 1.000 |  |  |  | 0.882 |
| 1 | 130 | 62 | 68 |  |  | 65 | 65 |  |
| ≥ 2 | 68 | 33 | 35 |  |  | 33 | 35 |  |
| Vascular invasion |  |  |  | 0.544 |  |  |  | 0.010* |
| Yes | 64 | 33 | 31 |  |  | 23 | 41 |  |
| No | 134 | 62 | 72 |  |  | 75 | 59 |  |
| Serum AFP level (ng/mL) |  |  |  | 0.445 |  |  |  | 0.022* |
| ≤ 200 | 136 | 68 | 68 |  |  | 75 | 61 |  |
| > 200 | 62 | 27 | 35 |  |  | 23 | 39 |  |
| Serum DCP level (mAU/mL) |  |  |  | 0.317 |  |  |  | 0.155 |
| ≤ 100 | 111 | 57 | 54 |  |  | 60 | 51 |  |
| > 100 | 87 | 38 | 49 |  |  | 38 | 49 |  |
| p53 |  |  |  | 0.071 |  |  |  | 0.072 |
| Positive | 50 | 18 | 32 |  |  | 19 | 31 |  |
| Negative | 148 | 77 | 71 |  |  | 79 | 69 |  |
| β-catenin |  |  |  | 0.864 |  |  |  | 0.302 |
| Positive | 43 | 20 | 23 |  |  | 18 | 25 |  |
| Negative | 155 | 75 | 80 |  |  | 80 | 75 |  |
