# Supplementary material for: Concurrent Activation of Acetylation and Tri-Methylation of H3K27 in a Subset of Hepatocellular Carcinoma with Aggressive Behavior
Source: PLoS One. 2014 Mar 10;9(3):e91330. doi: 10.1371/journal.pone.0091330 (PMC3948868; doi:10.1371/journal.pone.0091330)
Supplement: Table S2 — Multiple comparison in H3K27 modification groups using the log-rank test. (DOC) [file pone.0091330.s004.doc]

**Table S2.** Multiple comparison in H3K27 modification group using the log-rank test

|  | Overall survival | | | |  | Recurrence free survival | | | |
| --- | --- | --- | --- | --- | --- | --- | --- | --- | --- |
|  | H3K27 modification group | | | |  | H3K27 modification group | | | |
|  | A | B | C | D |  | A | B | C | D |
| A | - | 0.260 | 0.092 | 0.003** |  | - | 0.347 | 0.078 | 0.010 |
| B |  | - | 0.674 | 0.067 |  |  | - | 0.397 | 0.085 |
| C |  |  | - | 0.200 |  |  |  | - | 0.411 |
| D |  |  |  | - |  |  |  |  | - |

All figures are *P* values between two groups calculated with the log-rank test in the Kaplan-Meier method.

** *P* < 0.05/6 = 0.0083 (the Bonferroni adjustment)
